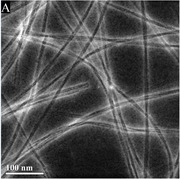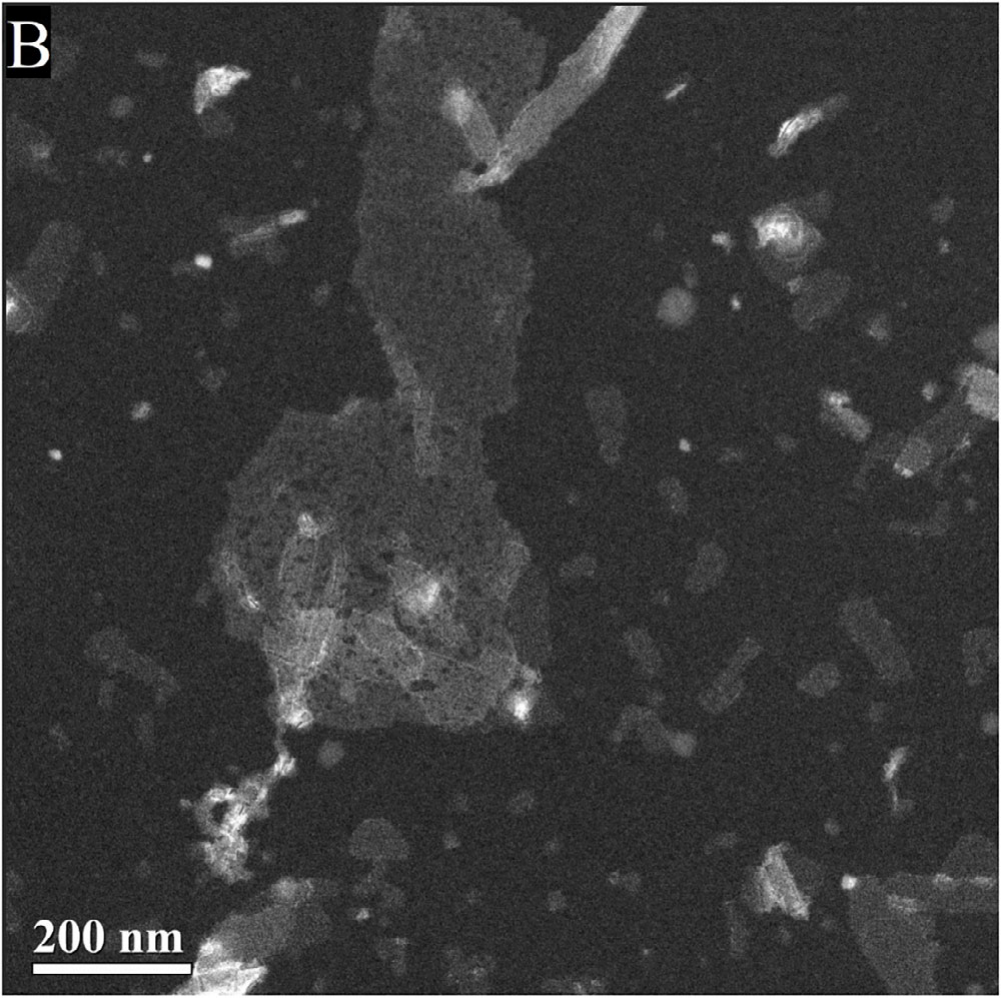# Microscopic Analysis of Triblock Heterochiral Peptide Assemblies.

**DOI:** 10.1002/alz70859_104128

**Published:** 2025-12-26

**Authors:** Jai Rudra, Dev Patel

**Affiliations:** ^1^ Washington University in St. Louis St. Louis, St. Louis, MO USA; ^2^ Early‐Career Researcher, Falls Church, VA USA

## Abstract

**Background:**

Autonomous organization, or self‐assembly of oligopeptides, with alternating polar and nonpolar structures, maintain the ability to assemble into fibrillar and soluble scaffolds, presenting an enormous opportunity for their use in combatting neurodegenerative diseases, such as Alzheimer’s. In nature, these organizational forces govern the self‐assembly of biomolecules such as lipid bilayers and DNA. It remains unquestioned that the adaptable nature of self‐assembly is reliant on the peptide’s chirality, or the position of the amino‐acids side chain. Herein, we examined the different uses of chirality in conjunction with L‐ and D‐ amino acids in primary peptide structures. Specifically, eight deblock heterochiral analogs of KFE12 peptides were examined and imaged using electron microscopy (EM).

**Method:**

KFE12 analogs were synthesized using solid‐phase peptide synthesis with the resulting resin being washed several times with Dichloromethane. The peptide then underwent a cleavage reaction and yielded a fluffy white precipitate of KFE12 after final filtration. 0.1mg is solubilized using ultra‐pure water and pasted onto EM grids and stained using uranyl‐formate. These grids are then placed into a dark environment until they are imaged using the JOEL 2100F Electron Microscope.

**Result:**

As portrayed, both peptides retain unique characteristics and properties concerning their structure. DLD can be seen to form ∼20nm wide fibrils or tapes and have a relatively consistent pitch of ∼200 nm. This morphology is unique in the fact that their assembly allows them to have observable twists when imaged. DLL has no consistent form of assembly and portrays “shrapnel” pieces with smaller peptide aggregates surrounding it. Instead of fibrils or tapes, DLL tends to form 2‐D β‐sheets, which may be attributed to the rapid depletion of the monomer phase of peptide assembly.

**Conclusion:**

Differing KFE12 peptides have been seen to produce different structures with unique properties depending on where the amino‐acids side chain lies. Though the mirror images (enantiomers) of some peptides may look similar between one another, infrared spectroscopy and circular dichroism spectroscopy has proved unique structure and folding and thus, unique properties. Further studies and imaging are needed to truly understand the mechanisms of self‐assembly and their clinical uses